# The Housing Instability Scale: Determining a Cutoff Score and Its Utility for Contextualizing Health Outcomes in People Who Use Drugs

**DOI:** 10.3390/ijerph22111653

**Published:** 2025-10-30

**Authors:** Fawaz Shanun, Daniel Jackson Smith, Beatrice King, Lydia Vlachou, Roesheen McGilvery, Stella Zine, Hayden Henderson, Emily Reichman, Nadiah Cunningham, Morgan Zare, Sarah Febres-Cordero

**Affiliations:** 1 Rollins School of Public Health, Emory University, Atlanta, GA 30322, USA; fawaz.shanun@emory.edu (F.S.); lydia.vlachou@emory.edu (L.V.); 2 School of Nursing, University at Buffalo, Buffalo, NY 14214, USA; dsmith55@buffalo.edu; 3 Opioid Program, Fulton County Board of Health, Atlanta, GA 30303, USA; beatrice.king1@dph.ga.gov; 4 Georgia Harm Reduction Coalition, Inc., Atlanta, GA 30314, USA; rmcgilv@emory.edu (R.M.); mojganz@ghrc-ga.org (M.Z.); 5 National Survivors’ Union, Greensboro, NC 27304, USA; stellapace7@gmail.com; 6 Positive Impact Health Centers, Decatur, GA 30030, USA; haydenvictoriah@gmail.com; 7 Nell Hodgson Woodruff School of Nursing, Emory University, 1520 Clifton Road NE, Room 220, Atlanta, GA 30322, USA; ereichma@asu.edu (E.R.); nccunni@emory.edu (N.C.)

**Keywords:** housing instability, people who use drugs (PWUD), drug-related health outcomes, cut-off

## Abstract

(1) Background: Housing instability, a key social determinant of health, disproportionately affects people who use drugs (PWUD), increasing their risk for adverse outcomes. This study explores the relationship between housing status and drug-related health outcomes among PWUD in an urban setting in the Southeastern United States (US) and determines the cutoff point for the Housing Instability Scale (HIS). (2) Methods: We conducted a cross-sectional survey from July to November 2024 among adult PWUD. Recruitment was through syringe services programs (SSPs), nightlife venues, and community outreach. HIS was used to assess housing status, while cluster analysis and Gaussian Mixture Modeling (GMM) were used to suggest a potential cutoff. Logistic regression models were employed to examine associations between housing instability and health outcomes. (3) Results: Among 164 participants (mean age = 41.2; 79.9% Black/African American), the average HIS score was 3.23. Cluster analysis suggested a singular cutoff, while GMM suggested four levels of housing instability. Multivariate logistic regression revealed that housing instability was significantly associated with infections (AOR = 1.55, *p* = 0.064), blackouts (AOR = 1.47, *p* = 0.0457), and seizures (AOR = 1.28, *p* = 0.0667). Overdose and wounds showed no significant association. SSP use, opioid use, and Xanax use were also identified as potential predictors.

## 1. Introduction

### Background

Housing stability is a fundamental determinant of health that impacts virtually every aspect of an individual’s life [1]. It is broadly defined as the lack of fixed or adequate housing, unstable neighborhoods [2], and homelessness due to the absence of a nighttime residence [3]. Being unstably housed has a greater impact on one’s health, including physical, mental, and overall well-being, compared to the general population [4,5,6]. A person who is unstably housed moves frequently, spends almost all their income on rent, or lives in a shared space that is grossly inadequate [7]. The most extreme version of housing instability includes unsheltered homelessness, in which people sleep in places that are not designed for human habitation, like sidewalks, train stations, tents, sheds, and garages [8].

Unstable housing has been associated with several adverse health outcomes, including obesity, cardiovascular diseases [9], mental disorders [10], infectious diseases like HIV, viral hepatitis [3,11], and limited healthcare access [12]. Furthermore, studies have shown that about 1 out of every 3 to 4 people experiencing homelessness use drugs [13,14]. People who use drugs (PWUD) and are experiencing homelessness suffer more harmful effects of drug use compared with people who are housed, including drug overdose-related deaths [15,16]. Individuals with substance use disorders and people who use drugs generally face multiple barriers to securing stable housing, primarily due to drug-related stigma and healthcare inequities [3,17].

The intersection of housing instability and drug use has become increasingly concerning amid the ongoing opioid crisis, with over 80,000 deaths recorded in 2022 alone—a surge largely attributed to the proliferation of synthetic opioids like fentanyl [15,16,18]. Infectious diseases like HIV/AIDS and Hepatitis C infections are a major cause of morbidity and mortality among people who inject drugs [19], and studies have found this association amplified among those experiencing homelessness or those who are unstably housed [6,20]. Identifying PWUD who are unstably housed or at imminent risk of homelessness is critical in implementing timely and effective public health interventions to prevent the negative impacts of drug use. Farero et al. have proposed the Housing Instability Scale (HIS) as a 7-item scale to track housing instability in both urban and rural populations [21]. The scale has been utilized in various forms in research. For example, Donoghue et al. (2025) used an abbreviated four-item version of the HIS among undergraduate students in a Hispanic-serving institution, finding it practical for identifying housing instability risk [22]. The full seven-item scale has been used in longitudinal studies such as those by Sullivan et al. (2023) and Goodman-William et al. (2023), who evaluate housing instability over a two-year period among survivors of the Domestic Violence Program [23,24]. Despite the diverse applications of this scale, there remains a lack of understanding of how the scale potentially categorizes the severity of housing instability, which could potentially aid in identifying individuals who would most benefit from public health interventions to support adequate housing.

The purpose of this study is to characterize the housing status of PWUD in an urban setting in the Southeastern United States (US) and has the following aims: (1) Examine the relationship between the housing status of PWUD and health outcomes (e.g., overdose, co-infections, wounds, seizures, and blackouts) and (2) propose a potential cut-off score of the HIS to categorize housing stability in PWUD.

## 2. Materials and Methods

### 2.1. Study Design

This cross-sectional study used a survey to collect data from people who use drugs to assess current drug use, housing insecurity, and health outcomes within a larger study focused on drug checking (e.g., fentanyl test strips, reagent kits). Recruitment occurred over five months in 2024 in an urban setting in the Southeastern US, using a convenience sampling approach through harm reduction syringe exchange programming, local nightlife events at bars and clubs, festivals, and snowball sampling. Eligible participants were adults aged 18 years and older who had used any unregulated drugs outside prescription in the past 12 months other than marijuana; there were no exclusion criteria. Of 180 people approached, 164 agreed to participate, yielding a study participation rate of 91%. The study protocol was approved by the Emory University IRB (STUDY00006583). Informed consent was obtained from all participants prior to data collection, but after the study purpose, the voluntary nature of participation, and privacy protections were explained. In addition, a Certificate of Confidentiality was obtained from the National Institutes of Health due to the criminalized nature of unregulated drug use, providing further protection to study participants.

### 2.2. Survey Development

The survey was developed in collaboration with community members with lived and living experiences of drug use and drug-related harm, harm reductionists, and researchers. Co-creation of such surveys is an important step to ensuring community priorities are included in studies [25] and is frequently seen in programs that involve PWUD [26,27]. Survey development was guided by the work of Maghsoudi et al.’s (2022) systematic review of drug checking services for people who use drugs [28]. Maghsoudi et al. (2021) identified 90 studies for review [28]. Health outcome concerns and consequences were identified as driving factors for drug checking in this study [28]. This knowledge, along with input from people with lived and living experience of drug use and health-related harm of drug use, guided the development of survey questions related to health outcomes. The survey collected self-reported data on demographics, housing status, substance use history, and drug-related health outcomes in the past 12 months. Self-reported past year occurrence of adverse drug-related outcomes was captured by the question, “Have you had any adverse effects suspected from drug use in the past year?” In addition to the options provided (e.g., overdose, co-infections, wounds, seizures, and blackouts), participants could also write in their responses. The frequency of adverse events was not collected. The housing status data were collected using the Housing Instability Scale [21].

### 2.3. Measure: The Housing Instability Scale

The Housing Instability Scale (HIS) is a seven-item validated scale that aims to measure housing instability to inform policies and public health interventions [21]. These seven items serve as indicators, dichotomized into a score of either 0 or 1. The higher the score (0–7), the more instability in housing. The specific items of the scale are:

1. Lived in an undesired or unstable housing situation in the past 6 months;
2. Uncertainty about maintaining current housing for the next 6 months;
3. Stayed with family or friends to avoid homelessness;
4. Experienced difficulty paying for housing (e.g., rent or mortgage);
5. Faced challenges obtaining stable housing;
6. Low confidence in ability to pay for housing this month;
7. Experienced frequent moving (3 or more times) in the past 6 months.

### 2.4. Data Management

All data were collected through RedCap [29] with restricted access to ensure participant confidentiality. Data was de-identified by assigning each participant an ID number to maintain confidentiality. Regular audits were conducted periodically to ensure data confidentiality and correctness.

### 2.5. Data Analysis

All analyses were conducted using R version 4.3.3 [30]. The following packages were used: dplyr [31], ggplot2 [32], corrplot [33], GGally [34], car [35], MASS [36], tidyr [37], psych [38], readr [39], sjPlot [40], tableone [41], flextable [42], officer [43], and mclust [44]. Descriptive statistics were calculated for demographic variables, housing scores, and patterns of drug use. Means and standard deviations were reported for continuous variables, and counts and percentages for categorical variables.

Drugs with similar mechanisms of action were grouped: The group ‘Opioids’ contained drugs like fentanyl, codeine, oxycontin, heroin, buprenorphine, nitazenes, and methadone. Methamphetamine and Adderall made up the group Amphetamine, while cocaine was used broadly for cocaine and crack use. The Housing Instability Scale (HIS) was used to assess potential housing instability in the sample [21]. Descriptive statistics were calculated for the housing instability score using the ‘dplyr’ package [32]. Logistic regression models were used to examine the association between housing stability and health outcomes. Both crude and adjusted models were estimated as follows:

- Crude models included housing stability as the sole predictor.
- Adjustment models controlled for relevant confounders such as SSP use, employment status, and use of specific drug classes (example: opioids, amphetamines).
- Model results were presented as Odds Ratios (OR) with 95% confidence intervals (CIs), along with corresponding *p*-values. Statistical significance was set at *p* < 0.05.

#### Cutoff Determination

Data visualization was conducted using the ‘ggplot2’ package, in which a bar plot and a kernel density plot were used to assess the shape of the distribution. The density plot was reviewed to assess where natural divisions in the housing instability score occurred [45]. To prepare the data for cluster analysis, scaling was performed to standardize the scores. K-means cluster analysis was performed using base R with k = 2, due to the appearance of two natural clusters in the data. This was confirmed using an elbow plot and 25 random starts to ensure model stability. Cluster assignments were visualized using density plots.

As a secondary validation strategy, we applied Gaussian Mixture Modeling (GMM) using the ‘mclust’ package. Model fit statistics, including Bayesian Information Criterion (BIC), were reviewed to assess model adequacy (Table A1). Review of BIC metrics showed that 7 clusters were optimal as determined by the lowest BIC. However, given that the HIS is a seven-point scale, we desired to assess groups within the scale. The next optimal number of groups was 4, 3, and 2 in descending order of BIC values, and all were within a magnitude difference of 10 of each other. Therefore, we chose G = 4 to balance model fit via the BIC and interpretability (compared to a four-group cluster). Subsequently, a three-component model (G = 4) was fit to the housing stability scale to estimate probabilistic classifications based on underlying normal distributions. Model summaries and classification plots were generated to visualize the results. All analyses were conducted on a complete-case dataset, excluding participants with missing values on any of the housing stability scale items.

## 3. Results

### 3.1. Demographic Characteristics

A total of 164 participants who use drugs in an urban setting in the Southeastern US, participated in this study. The average age of participants was 41.17 (SD = 13.8), with a bimodal age distribution. The minimum age was 18 and the maximum was 72. Of the 164 participants, 63.4% (*n* = 104) identified as male, 34.8% (*n* = 57) identified as female, and 1.8% (*n* = 3) chose neither. The majority of participants (79.9%, *n* = 131) were Black or African American, followed by White participants (15.9%, *n* = 26), while other racial groups constituted 4.2% (*n* = 7). Among participants, 35.3% (*n* = 49) were unemployed, 21.9% (*n* = 36) were fully employed, and 18.2% (*n* = 30) were self-employed. Additionally, 13.4% (*n* = 22) reported part-time employment, and 11.2% (*n* = 18) constituted other sources of income. The majority of participants (34.8%, *n* = 57) had completed high school or obtained a GED, followed by 23.8% (*n* = 39) who had some college education. Additionally, 17.7% (*n* = 29) had some high school education, and other levels of education constituted 11.6% (*n* = 19). 37 of the 164 participants (22.6%) reported using a syringe services program (SSP), while 76.4% (*n* = 124) of the study population did not. 3 (1.8%) individuals did not answer this question. Details are illustrated in Table 1.

### 3.2. Reported Drug Use

**Figure 1:**
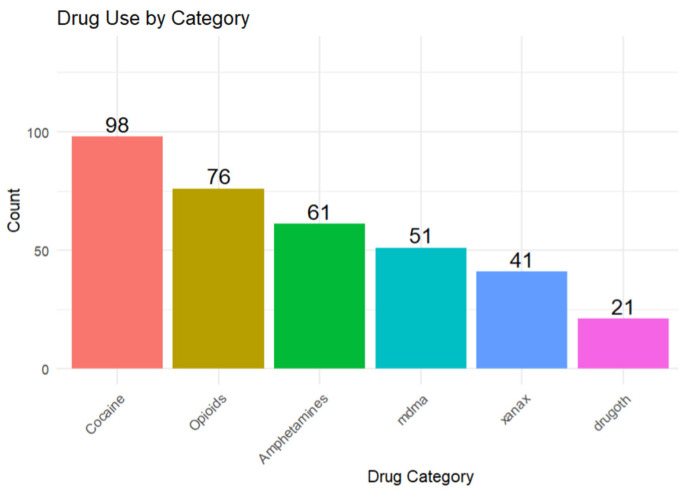
Bar Graph Displaying the Distribution of Drugs Used in the past 12 months.

The most commonly used drug was cocaine (59.8%, *n* = 98), followed by opioids (46.3%, *n* = 76). 37.2% (*n* = 61) of people used amphetamines, while about 31% (*n* = 51) used MDMA. 25% (*n* = 41) used Xanax, and about 13% (*n* = 21) of participants used drugs other than the ones mentioned above. The ‘other drugs’ category consists of drugs like mushrooms, ketamine, LSD, etc. Details are illustrated in Figure 1.

### 3.3. Housing Stability and Health Outcomes

Multivariate logistic regression analyses were conducted to examine associations between housing stability and various health outcomes among participants. Details are illustrated in Table 2.

### 3.4. Adverse Health Outcomes Results

Housing instability was significantly associated with increased odds of infection (AOR = 1.55, 95% CI: 1.16–2.21, *p* = 0.0064), blackouts (AOR = 1.47, 95% CI: 1.05–2.29, *p* = 0.0457) and seizures (AOR = 1.54, 95% CI: 1.09–2.37, *p* = 0.0257), but not with overdose or wounds, SSP use was consistently associated with higher odds of overdose (AOR = 4.04, 95% CI: 1.42–11.76, *p* = 0.009), infections (AOR = 3.92, 95% CI: 1.18–13.88, *p* = 0.0273), and wounds (AOR = 4.50, 95% CI: 1.38–15.80, *p* = 0.0142). Opioid use was significantly associated with infections (15.03, 95% CI: 2.67–284.90, *p* = 0.0121) and marginally with seizures (AOR = 6.77, 95% CI: 1.19–65.16, *p* = 0.0530), while Xanax use was linked to increased odds of seizures (AOR = 8.03, 95% CI: 1.84–43.35, *p* = 0.0078).

### 3.5. Housing Instability Scale Cut-Off

**Figure 2:**
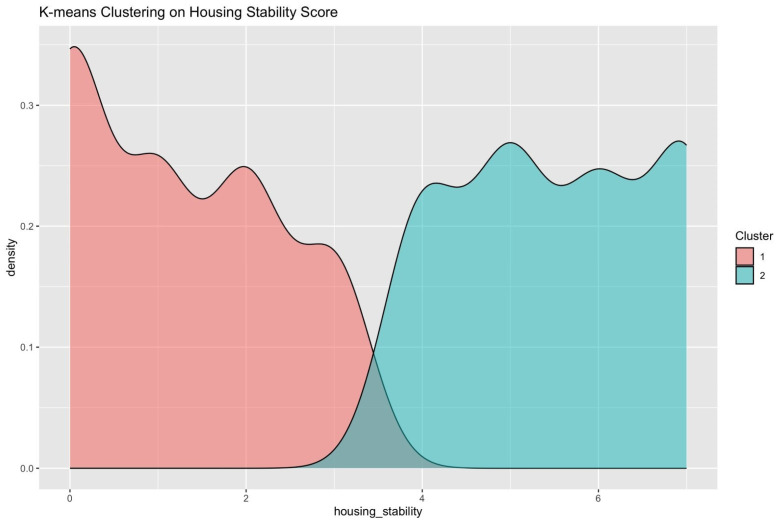
Distribution of Housing Stability Scores by Cluster Membership Identified via K-means Clustering. Cluster 1 represents those with low housing instability (scores 0–3), and cluster 2 represents those with high housing instability (scores 4–7).

**Figure A1:**
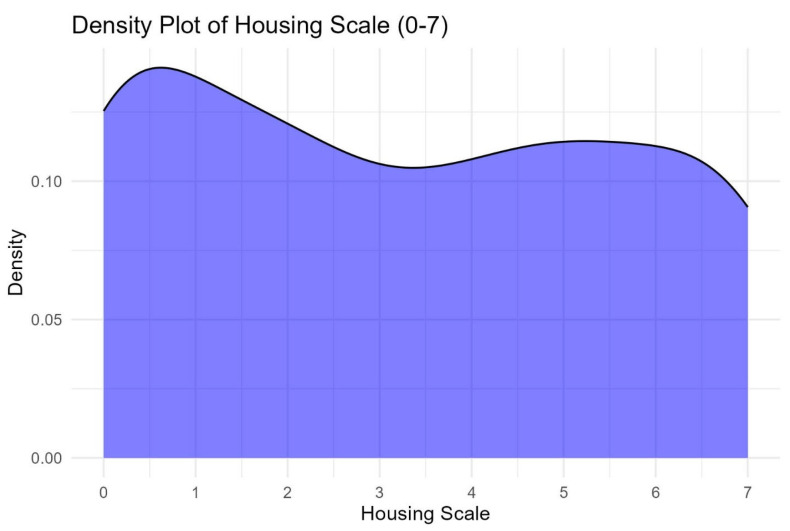
Density Plot of Housing Scale (0−7).

**Figure A2:**
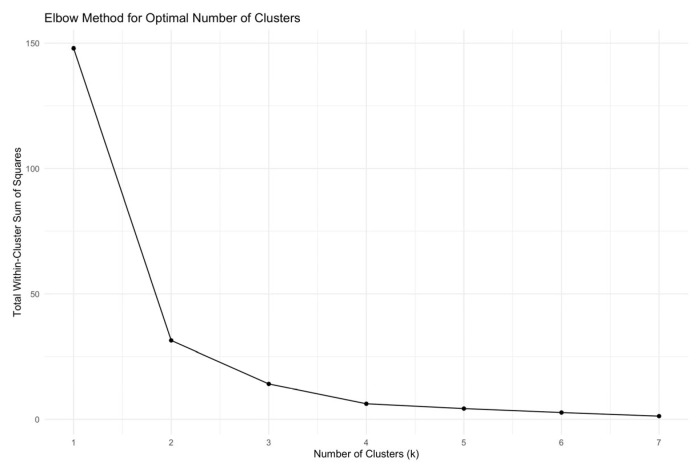
Elbow plot to determine optimal number of clusters in k-means clustering.

**Figure A3:**
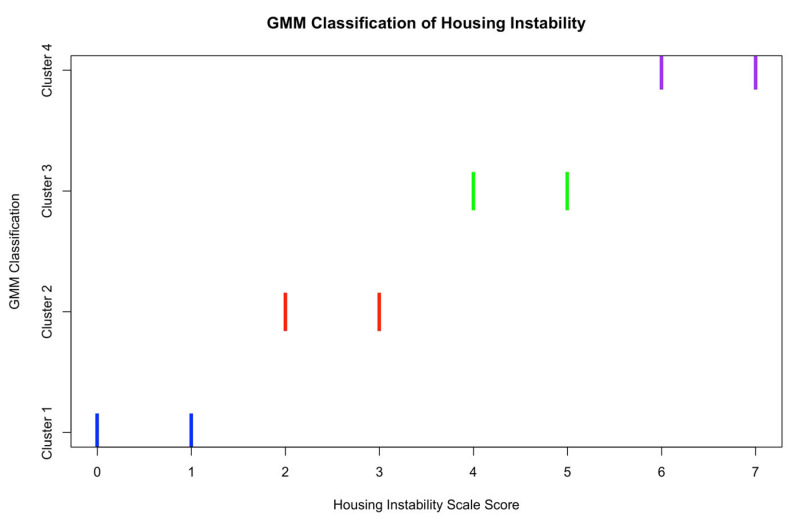
Classification of Housing Instability Scores Based on Gaussian Mixture Modeling. Cluster 1 represents those with high stability (scores 0−1), cluster 2 represents those with moderately high stability (scores 2–3), cluster 3 represents those with moderately low stability (scores of 4–5), and cluster 4 represents those with low stability (scores 6–7).

HIS scores range between 0 and 7, with higher scores indicating greater housing instability. The mean housing score overall for this study was 3.23 (SD = 2.43); SSP users had a higher score with a mean of 4.57 (SD = 2.34). The k-means cluster analysis suggested two potential clusters: cluster 1, representing those with scores of 0–3 (stably housed/stability) on the housing instability scale, and cluster 2, representing those with scores of 4–7 (unstably housed/instability) on the housing instability scale (Figure 2). This pattern was generally supported by the density plot (Figure A1) and elbow plot (Figure A2). Disparate results were found using GMM methodology, suggesting four potential classifications. Cluster 1, representing those with scores of 0–1 on the housing instability scale (high stability), and cluster 2, representing those with scores of 2–3 (moderately high stability), cluster 3, representing those with scores of 4–5 (moderately low stability), and cluster 4, representing those with scores of 6–7 (low stability) on the housing instability scale (Figure A3).

## 4. Discussion

This study aimed to characterize the housing status of people who use drugs in an urban setting in the Southeastern US, to examine the cut-off score for the HIS, and to examine the relationship between housing status and health outcomes, like infections, opioid-related overdoses, wounds, seizures, and blackouts. Our findings revealed significant associations between housing instability and the abovementioned adverse health outcomes. When confounders were adjusted for, housing status was not a predictor of overdose risk. However, it had a significantly positive association with the others: infections, wounds, seizures, and blackout risks.

### 4.1. Housing Instability and Health Outcomes

The relationship between housing stability and adverse health outcomes is well-documented. Housing instability has been reported to be associated with several health outcomes. A study conducted in Chicago found a significant positive association between housing instability and increased lifetime overdose count among people who inject drugs (PWID) [46]. This highlights the critical role that stable housing plays in protecting the health of vulnerable populations such as PWUD [47]. In our sample, higher housing instability scores appeared to correspond with increased odds of infections. Chiang et al. (2022) found that PWID who are unstably housed have an increased risk of infections, particularly HIV and HCV [48]. A systematic review conducted by Arum et al. (2021) also shows that PWUD who are unstably housed or experienced homelessness are 39% more likely to be diagnosed with HIV and have a 65% chance of HCV infection due to high-risk behaviors [3]. A global modeling study estimates that 17.2% and 19.4% of new cases of HIV and HCV, respectively, among PWID in high-income countries such as the U.S. are unstably housed [20]. It also suggests that individuals experiencing housing instability may be at elevated risk of engaging in health-compromising behaviors or experiencing conditions that predispose them to these adverse outcomes [3,49]. Beyond physical health, housing instability may expose individuals to increased risk of victimization, compounding mental health issues, which can further aggravate substance use outcomes [3].

Although housing instability showed a crude association with overdose, the adjusted model did not indicate a statistically significant relationship. This suggests that other variables, such as SSP use or unemployment, may also be at play. Cano et al. (2023) found that states with higher homelessness rates had higher overdose mortality rates across the United States [50]. Another study in Boston revealed that the leading cause of death among adults experiencing homelessness was overdose, with synthetic opioids accounting for 91% of those deaths [4]. Similarly, the association with wounds was not statistically significant. However, it has approached significance and may warrant further investigation with a larger sample.

#### 4.1.1. Role of SSP Use

Syringe Service Program (SSP) use emerged as a predictor in our sample across multiple health outcomes. Individuals who reported SSP use appeared to have higher odds of experiencing overdoses, infections, and wounds. However, results should be interpreted cautiously due to the wide confidence interval. This may support the notion that SSP users represent a high-risk subgroup of PWUD, some of whom may have started patronizing this service after experiencing complications such as infections from unsafe drug injection practices [51]. A study on a syringe service organization in Washington DC showed that 67% of its users were either homeless or unstably housed, with about a third reporting that their housing conditions impact their access to primary health care [52]. A systematic review conducted by Mackey KM et al. (2023) highlights that syringe exchange programs do not increase drug injection frequency but play a role in reducing the incidence of infections such as HIV and HCV, increasing naloxone education and possession, and linkage to care when necessary [53]. While SSP has been found to reduce the risk of HIV and hepatitis C, expanding the services to include wound care and the management of other skin infections could help address this gap [49].

#### 4.1.2. Substance Use as a Confounder

Certain drug use behaviors also contributed to adverse health outcomes in our sample. Opioid use in our sample was associated with infections and marginally associated with seizures in our study, highlighting its association with adverse health outcomes. This aligns with existing literature, which suggests that PWUD who are unstably housed are more likely to suffer from infections due to their limited access to healthcare, lack of water for personal hygiene, and care of injection-related wounds [47,54]. Additionally, despite a wide confidence interval, Xanax use appeared to be associated with seizures in our sample. This is biologically plausible as the abrupt withdrawal of benzodiazepines like alprazolam (Xanax) has been found to induce seizures, especially when combined with opioids [55].

#### 4.1.3. Housing Instability Scale Cut-Off

Housing is an independent determinant of health across all populations, regardless of drug use status [9,56,57]. Identifying a potential cutoff score for the HIS may provide a framework for more meaningful interpretation of the scale in research, similar to how tools like the PHQ-9 are used for depression screening [58]. However, further studies are needed to validate whether such cutoffs can reliably quantify housing instability as an independent determinant of health. In the context of this study, clinicians may benefit from having meaningful cutoff scores for the Housing Instability Scale (HIS) [21] to identify unstably housed patients, which would, in turn, help them understand disease patterns and curate preventive and targeted interventions for their patients based on housing insecurity. This is one of many reasons why establishing a validated cutoff score for the HIS is essential in the field of research, healthcare, and public health. Our study suggests possible cutoff ranges for the HIS as 0–1 (high stability), 2–3 (moderately high stability), 4–5 (moderately low stability), and 6–7 (low stability). Replication in larger and more diverse samples will be important to confirm their validity and practical utility.

#### 4.1.4. Public Health Impact

These study findings underscore the need for housing support among PWUD. The housing-first approach, which offers people housing regardless of their drug-use history, may be effective in reducing substance use and its health-related outcomes among PWUD [3,47]. Researchers, clinicians, and policymakers should support housing-first approaches when designing programs with PWUD due to the approach’s potential for success in reducing negative health effects associated with substance use. Importantly, the proposed cutoff for the HIS provides a practical tool to help identify individuals at the highest risk who could benefit most from such housing interventions. In addition, syringe service programs should expand to include primary healthcare, including wound care, to reduce the transmission and complications of infections, overdose education, and mental health services [49]. Lastly, this study indicated that unemployment was a predictor of an overdose. Economic empowerment through job training and integration could help address the root cause of drug-related harm and unstable housing. Implementing these comprehensive interventions that combine housing support and health services is crucial in improving health outcomes among this population.

#### 4.1.5. Limitations

There were several limitations in this study. Firstly, it was a cross-sectional study in a single urban area; therefore, causality cannot be determined. Repeating this study in other urban areas with PWUD and people who do not use drugs will help to further validate the HIS cutoff proposed here and the associations between housing and negative health outcomes due to substance use. Secondly, self-reported data on substance use may be subject to recall and social desirability bias. In future studies, the use of biomarkers to validate substance use and infections would enhance data accuracy. Thirdly, another limitation in this study is the sample size. The small sample size likely contributed to the wide confidence intervals observed in the analysis; therefore, results should be interpreted cautiously. Increasing the sample size in future research would enhance the precision of estimates, narrow confidence intervals, and improve the generalizability of findings.

## 5. Conclusions

This study contributes to understanding the complex interaction between housing stability and health outcomes among PWUD in an urban setting. While housing instability appeared to be associated with health outcomes such as infections, wounds, blackouts, and seizures, other predictors such as SSP use and unemployment were also found to be predictors. This underscores the multifaceted nature of housing insecurity and drug use that requires innovative approaches to intervention. These approaches include housing support, employment workshops for this population, and the expansion of syringe service programs to include primary healthcare. Addressing these structural determinants of health is essential for reducing disparities and improving health outcomes among PWUD in urban settings.

## Figures and Tables

**Table 1:** Baseline Characteristics of Participants (*N* = 164).

Characteristic	Level	Overall
Age, mean (SD)	—	41.72 (13.81)
Sex, n (%)	Male	104 (64.6)
	Female	57 (35.4)
Race, n (%)	Black or African American	131 (79.9)
	White	26 (15.9)
	Asian	2 (1.2)
	Other Race	3 (1.8)
	Unknown	2 (1.2)
Education, n (%)	High School Graduate or GED	57 (35.0)
	Some College	39 (23.9)
	Some High School	29 (17.8)
	Bachelor’s Degree	20 (12.3)
	Associate’s Degree	9 (5.5)
	Other	5 (3.1)
	Graduate Degree (Master’s, MD, PhD)	4 (2.5)
Employment, n (%)	Unemployed	57 (35.0)
	Employed full-time	36 (22.1)
	Self-employed	30 (18.4)
	Employed part-time	22 (13.5)
	Receiving disability	8 (4.9)
	Retired	6 (3.7)
	Unable to work due to disability/injury	2 (1.2)
	Other means of employment	2 (1.2)
Housing Stability, mean (SD)	—	3.23 (2.43)
SSP Use, n (%)	No	124 (77.0)
	Yes	37 (23.0)
Opioid Use, n (%)	No	81 (51.6)
	Yes	76 (48.4)
Cocaine Use, n (%)	No	61 (38.4)
	Yes	98 (61.6)
Xanax Use, n (%)	No	118 (74.2)
	Yes	41 (25.8)
MDMA Use, n (%)	No	107 (67.7)
	Yes	51 (32.3)
Amphetamine Use, n (%)	No	97 (61.4)
	Yes	61 (38.6)
Footnote: Percentages are calculated using the number of respondents with non-missing data for each characteristic; totals may not sum to 100% due to rounding.		

**Table 2:** Logistic Regression Results (Crude and Adjusted Odds Ratios).

Outcome	Predictor	Crude OR (95% CI)	Adjusted OR (95% CI)	*p*-Value
Overdose	Housing Stability	1.34 (1.10–1.66)	1.17 (0.94–1.48)	0.178
	SSP Use	–	4.04 (1.42–11.76)	0.009 *
Infections	Housing Stability	1.55 (1.16–2.21)	1.55 (1.16–2.21)	0.0064 *
	SSP Use	–	3.92 (1.18–13.88)	0.0273 *
	Opioid Use	–	15.03 (2.67–284.90)	0.0121 *
Wounds	Housing Stability	1.28 (0.99–1.69)	1.28 (0.99–1.69)	0.0667
	SSP Use	–	4.50 (1.38–15.80)	0.0142 *
	Opioid Use	–	1.77 (0.50–7.24)	0.392
Blackouts	Housing Stability	1.47 (1.05–2.29)	1.47 (1.05–2.29)	0.0457 *
	SSP Use	–	4.89 (0.74–98.00)	0.161
	Unemployment Benefits	–	4.38 (0.67–86.60)	0.188
Seizures	Housing Stability	1.54 (1.09–2.37)	1.54 (1.09–2.37)	0.0257 *
	Xanax Use	–	8.03 (1.84–43.35)	0.0078 *
	Opioid Use	–	6.77 (1.19–65.16)	0.0530

* Statistical significance *p* < 0.05.

## Data Availability

Data will not be shared publicly due to the sensitive nature of data collected from people who use drugs.

## Abbreviations

The following abbreviations are used in this manuscript:

United States	US
PWID	People who inject drugs
PWUD	People Who Use Drugs
SSP	Syringe Services Program
HIS	Housing Instability Scale
AOR	Adjusted Odds Ratio
OR	Odds Ratio
CI	Confidence Interval
MD	Doctor of Medicine
PhD	Doctor of Philosophy
GED	General Educational Development
R	R Programming Language (Statistical Computing), Version 4.3.3 was used for this analysis.
GMM	Gaussian Mixture Modeling
BIC	Bayesian Information Criterion
MPlus	MPlus Statistical Software
mclust	Model-based Clustering Package in R Version 4.3.3
HCV	Hepatitis C Virus
HIV	Human Immunodeficiency Virus
IRB	Institutional Review Board

## Appendix A

**Table A1:** Bayesian Information Criterion (BIC) values for Gaussian Mixture Models (G = 1–7). “E” and “V” refer to covariance parameterizations in the Gaussian Mixture Model. NA = model not available for that G.

Number of Components (G)	E	V
1	−696.59	−696.59
2	−669.51	−672.19
3	−668.72	NA
4	−659.09	NA
5	−669.33	NA
6	−672.18	NA
7	−632.07	NA
